# Medical and Physical Intelligence

**Published:** 1803-04-01

**Authors:** 


### MEDICAL AND PHYSICAL INTELLIGENCE.

### [ FOREIGN AND DOMESTIC. ]

Dr. Henning, of Zerbst, relates in his Contributions to Practical Medicine, several cases of hæmorrhagies, in which the phosphoric acid was successfully employed. The first time he tried this remedy, was in a lady thirty-one years old, of a full and plethoric habit, who had always suffered several irregularities in her menses, and was so extremely sensible and irritable as to be easily thrown into nervous fits. On the 22d of October, 1800, her menses came on, attended with different complaints, pains, &c. when a sudden fright excited convulsions, oppression in the chest, and congestions of blood in the lungs and the face, and the next day an hæmorrhagy of the uterus supervened. Mr. H. being sent for, ordered such remedies as are usually employed in this case ; but the state of the patient having become worse the next day, he had recourse to the phosphoric acid, of which ten drops in half a tea cup full of water were to be taken every hour. She had hardly taken the second dose, when the hæmorrhagy began to diminish, and before she had consumed half a drachm of the acid, it had entirely ceased. Dr. H. then administered a concentrated infusion of the Peruvian bark, to which he added half a drachm of phosphoric acid; of this mixture the patient took half a tea cup full four times a day, and by this means recovered within eight days.

A blacksmith of an athletic habit was attacked by an hæmoptisis, with stitches in the breast. After twelve ounces of blood had been taken from him, and a blister applied to the painful place, he was ordered to take ten or twelve drops of phosphoric acid every hour. The next morning the pains and hæmoptysis were entirely gone, and the man found himself able to return to his work.

In cases of direct asthenia of the vessels this remedy proves particularly efficacious, as will be seen from the following cases.

A pregnant woman, twenty-eight years of age, who had already had four children, and miscarried twice, was afflicted in consequence of a sudden fright, with an hæmorrhagy of the uterus, which threatened to cause a premature delivery. Mr. H. immediately gave the phosphoric acid from ten to fifteen drops every hour, and placed the patient in a horizontal situation. The pulse was small and slow. It is surprising, Mr. H. observes, what a quick and evident effect was produced here by two drachms of that acid ; all the alarming circumstances disappeared the next day, the patient found herself a little stronger, the pulse had risen, the appetite was better better, and she was in pretty good spirits. The phosphoric acid being in the same manner continued, she was restored to health within a few days, and at the proper time delivered of a healthy child.

A woman in childbed was attacked the third day after her delivery with a violent hæmorrhagy of the uterus, which required instant assistance. Dr. H. being called, found the patient cold, the face pale, pulse hardly perceptible, and the blood gushing out from the uterus. He immediately administered ten drops of phosphoric acid, and introduced a sponge, which was dipped in a mixture of one drachm of phosphoric acid and two tea cups full of water, into the vagina. The patient, who was almost half dead, recovered a little, after having for two or three hours continued the phosphoric acid: and though she was extremely weak the night after, she found herself much better in the morning; she had a quiet sleep for an hour, she was equally warm, and in a gentle perspiration; the lochiæ flowed in just quantity. She complained, however, of thirst, and was so extremely feeble, that the least exertion would make her swoon away; pulse weak. Some broth was given her from time to time, and the acid was continued every four hours. Being not worse the next day, she was ordered to take the following mixtures: ℞. Acid. phosphor. dr. ß. aq. cinamom. unc. iv. syrup. rub. idaei unc. j. D. S. every two hours a table spoon full to be taken. The next day he found the patient tolerably well, the hæmorrhagy had ceased, and the lochiæ were moderate; the breasts however were not yet filled with milk; pulse fuller and more regular; thirst diminished. A more nourishing diet was ordered, and a decoction of the bark prescribed. Towards the evening she was restless, complained of head-ach, shivering, tension under the arms; lochiæ were more copious, the belly extended, &c. all which symptoms however Dr. H. derived from the secretion of the milk. The next morning, after having much perspired, she found herself more easy, and the breasts began to be filled. The medicines were continued. Thus the patient grew gradually better, the lochiæ diminished, the fever had ceased, and after eight days she entirely recovered.

The phosphoric acid is, above all, of singular service in scorbutic hæmorrhagies, of which Dr. H saw two or three instances where it effected a cure, after other remedies had been employed without success.

---

M. Dubois, Principal Surgeon to the Hospital of the Medical School, (Ecole de Perfectionment ci devant St. Come) has applied moxas with success in two cases of loss of speech after fever. The patients had lost entirely any power over these organs for a very considerable time. In one case, the speech returned after the second application to the back of the neck, or rather between the shoulders. In the other, though the effects of the application were manifest on the part, yet the person was not compleatly cured till after a third application. The modus operandi of these remedies is by effecting the nervous excitement which they induce.

---

M. Bosquillon, Docteur Regent of the ci-devant Faculty of Medicine of Paris, Ancient Professor of Surgery and Materia Medica, has lately published a Memoir to prove that Hydrophobia is a disease incapable of being communicated; in short, that it is a disease without any real existence. This he endeavours to prove, in part, by the History of Medicine: He finds no mention made of it in the works of Hippocrates; Aristotle says it is a kind of phrenzy peculiar to the dog, and capable of being communicated to every animal except man. Nuardin, in a poem on the subject of poisonous animals, does not notice it; Plutarch speaks of it as a new disease. If canine madness be a disease which at all times existed, but which was originally not communicable to man, some change must have taken place in his constitution. Dr. B. brings some facts, unnecessary to state, in order to prove that this is merely a kind of mania, a conceit, the result of apprehension from having been bitten. There is a vulgar saying, that conceit is as bad as consumption, if it kills; and we may say, in this case, that it is as bad as Hydrophobia. The arguments to disprove this opinion of Dr. B. will dart on every mind.

---

Mr. Pepys, jun. has lately constructed the most powerful Galvanic apparatus that has been yet produced. It consits of 60 pair of zinc and copper-plates, disposed in two troughs, constructed on Mr. Cruikshank's plan, but with some accompanying arrangements which are extremely convenient and useful. The experiments made with this apparatus by Mr. Pepys, on the deflagration of metals, were the most brilliant and splendid ever beheld in London; of which the following account will give some idea: The troughs were filled with 32 pounds of water, mixed with two pounds of concentrated nitrous acid. With this charge, iron-wires of $\frac{1}{250}$ to $\frac{1}{10}$ of an inch in diameter were deflagrated with great splendour. A number of the small ones, twisted together, produced somewhat like a little brush deflagration.—Charcoal of box-wood was not only deflagrated at the place of contact, but remained permanently red-hot for near two inches in length.—Lead-foil burnt with great vividness, becoming red-hot, and emitting a small volcano or adjutage of red sparks with the flame.—Tin-foil burnt with great splendour, with smoke and sparks.—Dutch leaf or brass-foil deflagrated vividly, with smoke and a profussion of sparks.—Silver-leaf burnt with an intense vivid green light.—Gold-leaf deflagrated with a white bright light.—Tin-wire $\frac{1}{5}$ of an inch in diameter, fused, burnt, and oxidated, with great splendour.—Platina-wire $\frac{1}{16}$ of an inch in diameter, became red-hot, white, and fused in globules at the contact.—Gunpowder, phosphorus, and inflammable substances, are instantly fired by contact with conductors armed with charcoal.—The Galvanic power was capable of deflagrating charcoal, charcoal, after passing through sixteen persons with wetted hands joined.

---

### LIST of the OFFICERS and COUNCIL of the MEDICAL SOCIETY of LONDON, for 1803.

PRESIDENT.

1 Dr. James Sims

TREASURER.

1 Dr. Sayer Walker

LIBRARIAN.

1 Mr. Hurlock

SECRETARIES.

1 Dr. Robert Hooper
2 Mr. Aikin

SECRETARY FOR FOREIGN CORRESPONDENCE.

1 Dr. Marcet

### COMMITTEES.

I. THEORY AND PRACTICE OF PHYSIC.

1 Sir J. M. Hayes, Bart.
2 Dr. Hulme
3 Dr. Lettsom
4 Dr. Saunders
5 Dr. Bradley

II. ANATOMY AND PHYSIOLOGY.

1 Dr. Jenner
2 Mr. Andree
3 Mr. Abernethy
4 Mr. Astley Cooper
5 Mr. Macartney

III. SURGERY.

1 Mr. Ford
2 Mr. Norris
3 Mr. Ware
4 Mr. Forster
5 Mr. Blair

IV. MIDWIFERY.

1 Dr. John Sims
2 Dr. Poignand
3 Mr. Ridout
4 Mr. Webb
5 Mr. Ring

V. MATERIA MEDICA AND PHARMACY.

1 Dr. Yelloly
2 Dr. Curry
3 Mr. Jackson
4 Mr. Field
5 Mr. Spry

VI. BOTANY AND NATURAL HISTORY.

1 Dr. Elliott
2 Dr. Rogers
3 Dr. Pearson
4 Dr. Dimsdale
5 Mr. Chamberlaine

VII. NATURAL PHILOSOPHY AND CHEMISTRY.

1 Dr. Babington
2 Dr. Pinckard
3 Mr. Parkinson
4 Mr. J. H. Hooper
5 Mr. Seaton

ANNIVERSARY ORATION FOR NEXT YEAR.

1 Dr. Haighton

REGISTRAR.

1 Mr. Charles Aikin

---

A letter from Capt. KEYT, of the 51st regiment, dated Columbo, September 23, 1802, to Mr. EATON, of Halton, contains the following paragraph:—“ Within a few days vaccine virus has arrived in Ceylon, from Bombay, on small threads; and one Cyngalese woman woman has taken the infection by inoculation. Mr. North, the governor, is endeavouring to extirpate the small-pox, by inviting the natives to undergo the operation of inoculation with vaccine virus."

It is with pleasure we can inform our readers, that Mr. Wilkinson's work on the Broad-leaved Willow Bark, with a coloured plate of the same, is in great forwardness, and will be published some time in the spring of the present year.

Dr. Jameson, of Bloomsbury, is writing a Treatise, which will be published next month, entitled An Enquiry into the Effects of Climate upon the Human Body, to discover the causes of the Catarrhal Epidemic which has prevailed this Spring, and in former years, under the name of Influenza.

